# Hysteresis Analysis and Positioning Control for a Magnetic Shape Memory Actuator

**DOI:** 10.3390/s150408054

**Published:** 2015-04-07

**Authors:** Jhih-Hong Lin, Mao-Hsiung Chiang

**Affiliations:** Department of Engineering Science and Ocean Engineering, National Taiwan University, No.1, Sec.4, Roosevelt Rd., Taipei 106, Taiwan; E-Mail: d98525020@ntu.edu.tw

**Keywords:** magnetic shape memory alloys, magnetic shape memory actuator, modified fuzzy sliding mode control, hysteresis, positioning control

## Abstract

Magnetic shape memory alloys (MSM alloys), a new kind of smart materials, have become a potential candidate in many engineering fields. MSMs have the advantage of bearing a huge strain, much larger than other materials. In addition, they also have fast response. These characteristics make MSM a good choice in micro engineering. However, MSMs display the obvious hysteresis phenomenon of nonlinear behavior. Thus the difficulty in using the MSM element as a positioning actuator is increased due to the hysteresis. In this paper, the hysteresis phenomenon of the MSM actuator is analyzed, and the closed-loop positioning control is also implemented experimentally. For that, a modified fuzzy sliding mode control (MFSMC) is proposed. The MFSMC and the PID control are used to design the controllers for realizing the positioning control. The experimental results are compared under different experimental conditions, such as different frequency, amplitude, and loading. The experimental results show that the precise positioning control of MFSMC can be achieved satisfactorily.

## 1. Introduction

Smart materials are materials for which some properties can be repeatedly controlled by external conditions, such as temperature, electricity, and stress. Over the past two decades, a new smart material, magnetic shape memory (MSM) alloy, has been developed. Its length can be changed by subjecting it to a magnetic field and mechanical stress. Additionally, up to 6%–10% of the strains can be induced by an external magnetic field, and this property of MSM alloys has attracted the interest of researchers [1,2,3,4,5]. MSM alloys also have the advantage of fast response [4]. Some smart materials, like piezoelectric ceramics, magnetostrictive materials, and shape memory alloys, can change their length by the adjustment of external conditions. Compare these smart materials with MSM alloys, piezoelectric ceramics, and magnetostrictive materials, which have fast response but small strokes, and shape memory alloys bear larger strain but have a slower response. Therefore, all of these properties give MSM alloys the potential to be applied in many different fields in the future.

**Figure 1:**
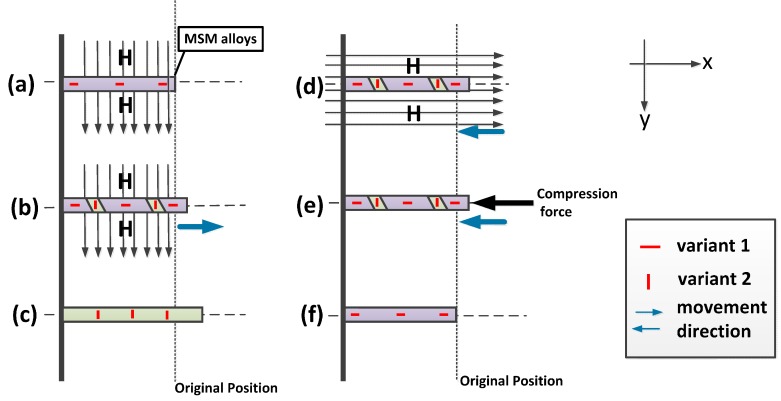
Operation of MSM alloys. (**a**) MSM alloy has only variant 1 in the first state; (**b**) With the magnetic field, the MSM alloy is extending; (**c**) Without the magnetic field or when the field is small, the MSM alloy retains its length; (**d**) MSM alloys can be contracted by providing another direction for the magnetic field; (**e**) MSM alloys can be contracted by providing compression force; (**f**) Through (**d**) or (**e**), MSM alloys can return to their first state.

There are several composites for MSM alloys, such as Fe-Pb, Fe-Ni, and Ni-Mn-Ga [6,7]. Ni-Mn-Ga is mainly used because of its large induced strain under a magnetic field. MSM alloys have two phases: austenite in high temperature and martensite in low temperature. MSM alloys have the same properties as shape memory alloys, which can work their shape memory effect between martensite and austenite [8]. In addition, MSM alloys have a magnetic shape memory effect in the martensite phase, which is composed of twin variants (here we call them variant 1 and variant 2). The magnetic shape memory effect is applied in this paper. The operation of MSM alloys is shown in Figure 1. Originally, MSM alloys have only variant 1, as in Figure 1a. If MSM alloys receive a magnetic field in the direction of axis y, then MSM alloys will be extended in the direction of axis x as in Figure 1b. The cause of elongation is the transformation of martensite twin-variants. Variant 1 and variant 2 are lined in different specific directions. When the magnetic field passes through MSM alloys, it causes variant 1 to turn to variant 2 [4]. The elongation of MSM alloys will be saturated depending on the strength of magnetic field. When the magnetic field is turned off or MSM alloys are saturated under the specifically magnetic field, both of them could not drive more variant 1 to variant 2; then, the MSM alloys retains the length of its elongation as in Figure 1c. The way to compress the MSM alloys is to turn variant 2 to variant 1. This could be done by providing a magnetic field in the direction of x-axis as in Figure 1d or providing compression stress as in Figure 1e, such as by using a spring, to make variant 2 turn back into variant 1, as shown in Figure 1f [9].

MSM alloys also have some drawbacks: its nonlinear behavior of wide hysteresis and the strong influence of temperature [10]. Many research groups have developed mathematic models for describing the magnetic field-induced strain of MSM alloys. Likhachev and Ullakko [11] considered the thermodynamics of the mechanical and magnetic properties of MSM alloys. Gauthier *et al.* [12,13] provided models for chemical energy, thermal energy, mechanical energy, and magnetic energy, to explain the rearrangement process between martensite variants in an MSM alloy. For the purpose of controlling the MSM actuator, Tan and Elahinia [14] combined the physical models with whole system models, including kinematics of MSM actuator and electromagnetic model. However, physical models are complex since they need to concern all the factors that can influence the variants of MSM alloys. Riccardi *et al.* [15,16] treated the whole MSM actuator as a series of linear dynamic components and a hysteretic nonlinearity. A modified Preisach model was the way to describe the hysteretic nonlinearity, and adaptive control was used to provoke cancellation of the hysteresis. Hysteresis compensation is not only seen in the Preisach model; phase shift hysteresis compensation was also used by Sadeghzadeh *et al.* [17]. PID with gain scheduling was used as the position controller.

The MSM alloy can work as an actuator and a sensor just as in piezoelectric actuators. If the input magnetic field is given, the MSM alloy can serve as an actuator to generate displacement and force. By contrast, if an external disturbance force is inserted to the MSM alloy, the magnetic field will change. Therefore, the MSM can also be used as a sensor for measuring the magnetic field [18,19]. This paper concentrates on the characteristics and control of the MSM actuator.

Fuzzy control has been widely used in engineering. Since fuzzy methodology is a promising new method to control engineering without considering the complexity of chemical phase change, we could ignore the construction of the mathematical model of the phase change. It is suitable as a control, especially in variants transforming of MSM alloys, where the model would be involved in many facets, such as variant transform in MSM, temperature, dynamics, and the hysteresis phenomenon. In addition, sliding mode control has the advantage of robustness; it will reach stability a limited time after entering the sliding surface. Therefore, fuzzy sliding mode control (FSMC) can achieve robustness and a model-free controller. Only the step response under no-load conditions, directly controlled by FSMC, was discussed in Chiang and Lin [20]; we found some problems with that approach. The position control error will approach zero in the steady state, and the control signal of direct FSMC will also approach zero such that the MSM actuator cannot keep the desired position and become zero position output. Thus, in this paper the modified fuzzy sliding mode control (MFSMC) is proposed by adding an additional integrator in FSMC for further modification of controller, and additional experimental investigations and analysis are performed, such as the further improved position accuracy of the step response and the path control of sinusoid trajectory with 100 g load, under different amplitudes and frequency.

The purpose of this paper is to analyze the hysteresis and develop a closed-loop positioning control for a MSM actuator. Section 2 introduces the overall system of the MSM actuator in this paper. Section 3 explains the concept of fuzzy sliding mode control and the proposed modified fuzzy sliding mode control. Section 4 shows the results of experiments, including the open loop test of wide hysteresis of MSM alloys and closed-loop control of step response and position tracking of sine. The experimental results show that modified fuzzy sliding mode control can achieve better position accuracy than PID control. Finally, Section 5 is the conclusion for this paper. 

## 2. MSM Actuator Overview

### 2.1. Mechanism Description 

**Figure 2:**
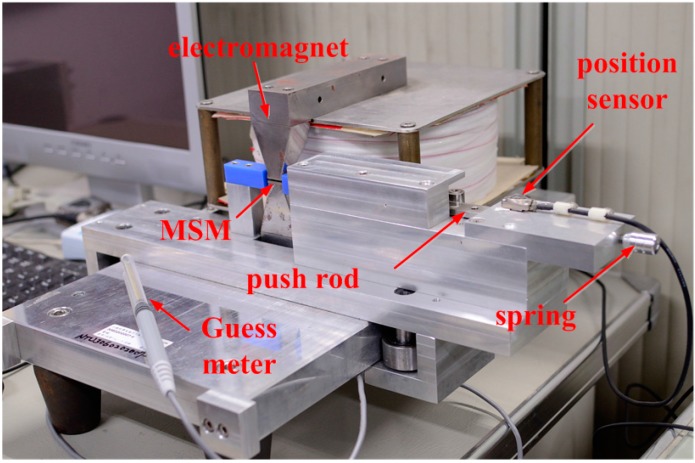
Photograph of the MSM actuator.

The photograph of the MSM actuator is shown in Figure 2. The MSM actuator consists of a magnetic circuit, MSM elements, and a returning force spring. The magnetic circuit includes the coils and the flux guide. The flux guide material is made of pure iron. The MSM element is a piece composed of five M Ni-Mn-Ga alloys [21]. The dimensions of the MSM element is 20 mm × 2.5 mm × 1 mm in size, and it can generate movement of elongation from magnetic excitation. In the air gap of the magnetic circuit, the longest edge of the MSM element material is placed along the transversal direction (x-axis), and the magnetic field passes through the MSM element in the vertical direction (y-axis). The MSM element will be elongated when the magnetic field is provided. The returning force spring can contract the MSM element, since the MSM element has inherent holding power even when the magnetic field is turned off. The returning force can be generated in two ways. It can be provided by a magnetic field to be parallel with the MSM element (x-axis), or it can be given by a spring. The return force spring is used in this paper, and a push rod is used between the spring and the MSM element for fitting sensors.

### 2.2. Test Rig Layout

**Figure 3:**
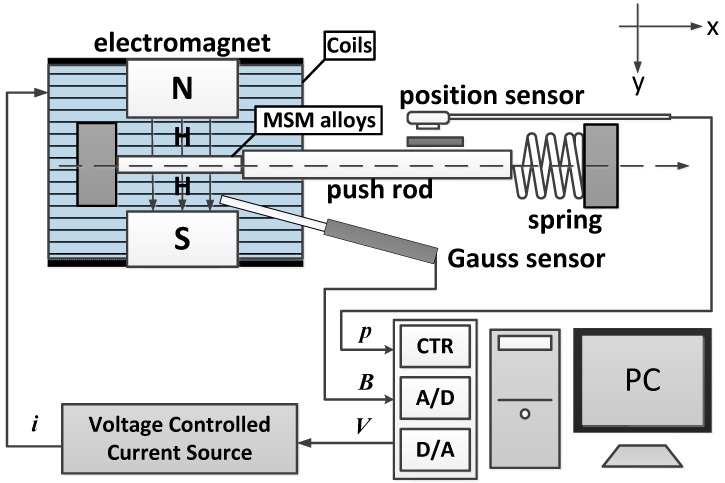
Structural illustration of MSM actuator.

Figure 3 shows the overall system of the MSM actuator. The magnetic field is provided by a C-shaped electromagnet. An optical linear encoder with 20 nm resolution is used for measuring the position of the MSM actuator movement. A Hall probe is inserted into the air gaps for measuring the flux density. All the signals are fed back to a PC-based controller via a multifunction DAQ card. The sampling frequency is 1000 Hz.

Real Time Windows Target developed by Mathworks Ltd. (Natick, MA, USA) was used for achieving the control system. It enabled us to create a real-time control system for hardware-in-the-loop simulation. Real Time Windows Target was utilized to automatically generate C codes and executable files from this Simulink model. The generated executable file runs in real time on the personal computer with 1 ms of sampling time and realizes a real-time control system. This allows for easy design and rapid testing of the control algorithms with the actual hardware. When running controlled experiments, the multifunction DAQ card gives voltage (0–1 V) to the voltage controlled current source, then electric current will be provided at the rate of 1.35 A/1 V in a linear relationship. The advantage of the electromagnet is that it needs only a small electric current to create a strong magnetic field. We only need to give an electric current of 0.7 A to the system for the magnetic field to reach almost 1 Tesla. The saturation-induced strain of the MSM element is around 0.9 Tesla. The magnetically-induced strain of the MSM element can be easily affected by the temperature change [7]. In this way, small electric current can reduce the increase of the temperature in the experiments, so that the temperature can be maintained at a constant value. Table 1 shows the specifications of the test rig.

**Table 1:** Specifications of test rig.

Components	Specifications
PC-based controller	CPU Pentium 4; 16 bits ADC × 16 CH; 16 bits DAC × 2 CH; Digital I/O × 24 CH; 32 bits counter × 2 CH
Power supply	Power: 360 W; Voltage range: 0~80 V; Current range: 0~13.5 A; Voltage Controlled Current Source mode: 1.35 A/1 V
Gauss sensor	Range: 0.000~±3.000 Tesla; Frequency Range: DC and 15 Hz to 10 kHz; Analogue Output: ±3 V full scale
Optical encoder	Range: 25 mm; Resolution: 20 nm
Spring	Spring Constant: 3.5 N/mm
MSM element	Element size: 20 mm × 2.5 mm × 1.0 mm Large strain: typically 3%–5%, up to 6% Max. magnetic field 700 mT

## 3. Modified Fuzzy Sliding Mode Control Concept

### 3.1. Modified Fuzzy Sliding Mode Control Theory 

**Figure 4:**
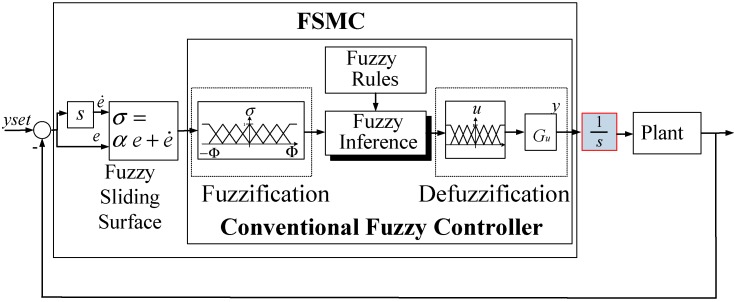
Block diagram of MFSMC system.

Fuzzy control theory involves fuzzification, a fuzzy rule base, fuzzy inference, and defuzzification. The fuzzy rules base, which is generalized from experts’ experience, however, is sometimes unsuitable because of wrong experiences or overly complex membership functions. The stability and the robustness are difficult to guarantee. Fuzzy sliding mode control (FSMC) theory is one of the means to tackle this problem [22,23,24]. In many fuzzy control systems, the fuzzy rule base simultaneously depends on both control error *e* and error rate $\overset{\cdot }{e}$ such that the number of the fuzzy inference rules increases and the membership functions are complicated. The fuzzy sliding mode control system, however, introduces a fuzzy sliding surface function *σ* for reducing the dimensions of the input space and the number of fuzzy inference rules. The stability and the bound of the tracking error can also be guaranteed by using Lyapunov’s theory. In order to make the MSM actuator keep the desired position as the position error approaches zero, the modified fuzzy sliding mode control (MFSMC) is proposed by inserting an integrator. Figure 4 depicts the block diagram of the modified fuzzy sliding mode control system. 

### 3.2. Designing MFSMC for Positioning Controller

**Figure 5:**
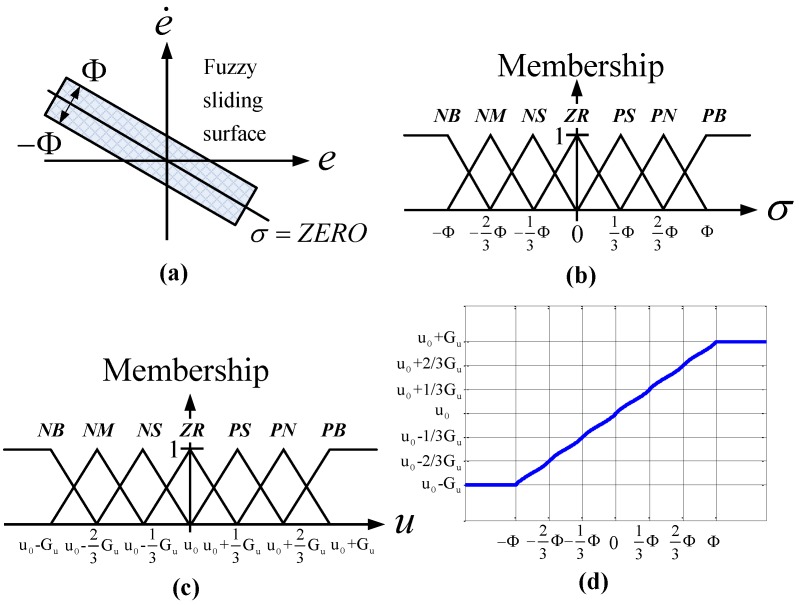
Fuzzy sliding surface and rule base of MFSMC: (**a**) fuzzy sliding surface σ, (**b**) membership function of fuzzy sliding surface σ, (**c**) membership function of control input *u*, and (**d**) defuzzified control input *u_f_*.

Consider a non-linear system

(1) $$x^{\left(n\right)}(t)=f(x,t)+d(t)+u,\;x^{\left(n\right)}=\frac{d^{n}x}{dt^{n}}$$

where $x(t)={\left[x,\dot{x},...,x^{\left(n-1\right)}\right]}^{T}$ is the state vector, *f*(**x**,*t*) is a non-linear function with an bound of **F**(*x*,*t*), *d*(*t*) is a disturbance bounded by **D**(*t*), and *u* is the control input. The tracking error of the state vector is defined as

(2) $$e(t)=x(t)-x_{d}(t)={\left[e,\dot{e},...,e^{\left(n-1\right)}\right]}^{T}$$

where $x_{d}(t)$ is the desired output. The fuzzy sliding surface is introduced to simplify the fuzzy rules:

(3) $$\sigma (x,t)={\left(\frac{d}{dt}+\alpha \right)}^{n-1}\;e=0$$

where α is a positive constant [25]. In the fuzzy sliding mode control system, the fuzzy sliding surface of the second order system, as shown in Figure 5a, is

(4) $$\sigma =(\dot{e}+\alpha \;e)=ZERO$$

where the positive constant α is the gradient of the fuzzy sliding surface σ=ZERO, which is a straight line in the phase plane. Φ indicates the boundary layer of the fuzzy sliding surface σ. Figure 5b,c show the membership functions of the sliding surface σ and the control input *u*. The fuzzy sliding surface σ is divided by the membership function set M(σ)={NB,NM,NS,ZR,PS,PM,PB}, where NB, NM, NS, ZR, PS, PM, and PB are negative big, negative medium, negative small, zero, positive small, positive medium, and positive big. The control input $u_{f}$ is partitioned by the membership function set, M(u)={NB,NM,NS,ZR,PS,PM,PB}. Therefore, from the 7 × 7 fuzzy rules that depend on control error *e* and error rate $\dot{e}$ in the conventional fuzzy control, the fuzzy sliding mode requires only seven fuzzy rules, using the fuzzy sliding surface σ. Thus, the fuzzy rule base can be simplified as Table 2.

The Mamdani method is used for fuzzy inference and the center-of-area defuzzifier is used for defuzzification:

(5) $$u_{d}=G_{u}(x,t)\cdot \frac{\int \mu (u)\cdot u\cdot du}{\int \mu (u)\cdot du}$$

where *µ*(*u*) indicates the membership function of *u*. Figure 5d depicts the results of the control input *u_f_*, which can be described as

(6) $$u_{f}=u_{o}-u_{d}$$

However, as the position control error approaches zero in the steady state, the control signal of FSMC will also approach zero such that the MSM actuator will become zero position output. Thus, this paper proposed the MFSMC. By the additional integrator the control signal can be maintained in a steady output value as the position error approaches zero in the steady state, such that the MSM actuator can keep the desired position.

**Table 2:** Fuzzy rules.

Rules	Description
${\text{R}}^{1}$	IF σ is PB THEN u is PB
${\text{R}}^{2}$	IF σ is PM THEN u is PM
${\text{R}}^{3}$	IF σ is PS THEN u is PS
${\text{R}}^{4}$	IF σ is ZR THEN u is ZR
${\text{R}}^{5}$	IF σ is NS THEN u is NS
${\text{R}}^{6}$	IF σ is NM THEN u is NM
${\text{R}}^{7}$	IF σ is NB THEN u is NB

## 4. Experiments

In this section, the experiments of the MSM actuator in the open loop test and in the closed-loop control are presented. In the open loop test, the hysteresis phenomenon of MSM actuator with different input signals is analyzed. In the closed-loop control, the MSM actuator is controlled and compared by the PID control and the MFSMC under different path profiles and loading conditions.

### 4.1. Hysteresis Test

**Figure 6:**
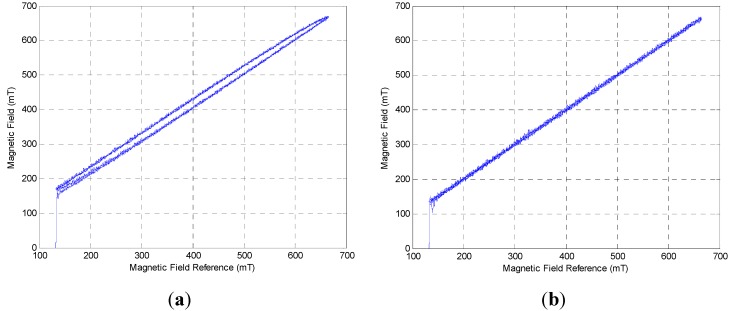
(**a**) Output of magnetic field in open loop system; (**b**) Output of magnetic field with PID control.

For testing the hysteresis phenomenon in the MSM actuator, the data when the current increased linearly from 0 A to 0.6 A, and *vice versa*, are provided. In Figure 6a, it is shown that the electromagnet also has a hysteresis phenomenon. Therefore, a PID control is used to reduce the influence of the electromagnet and linearize the output magnetic field, as shown in Figure 6b.

**Figure 7:**
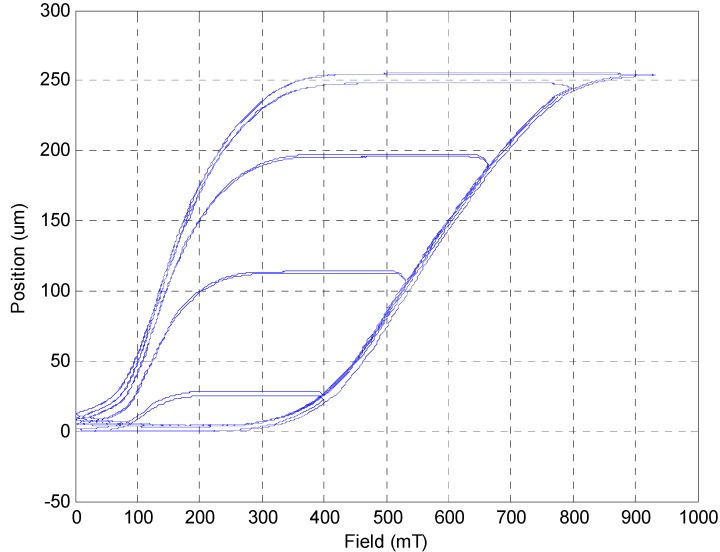
Different loops of hysteresis of the MSM actuator.

Figure 7 shows different loops of hysteresis, including the major hysteresis loop and the minor hysteresis loop, caused by different magnetic field output. The threshold of the magnetic field is around 300 mT. The major hysteresis loop shows that the saturation of MSM actuator is around 900 mT. When the strength of the magnetic field decreases, it still needs to be under around 350 mT so that the MSM actuator can be compressed by the returning force from the spring. Thus, the MSM actuator obviously has a wide hysteresis phenomenon.

### 4.2. Step Response in Closed-Loop Control

**Figure 8:**
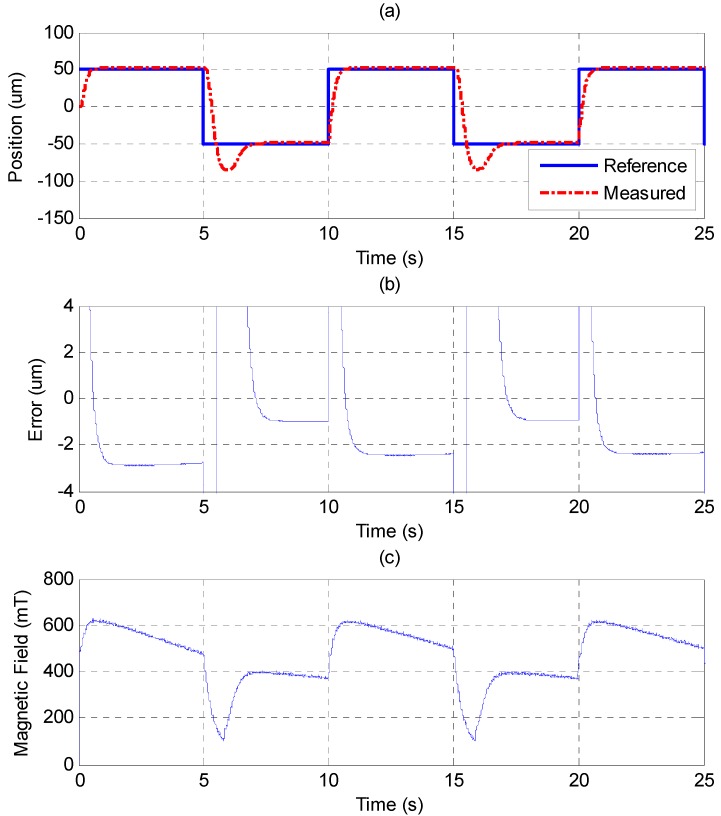
Step response of ±100 μm by PID controller: (**a**) position control response; (**b**) control error; (**c**) control input (magnetic field).

**Figure 9:**
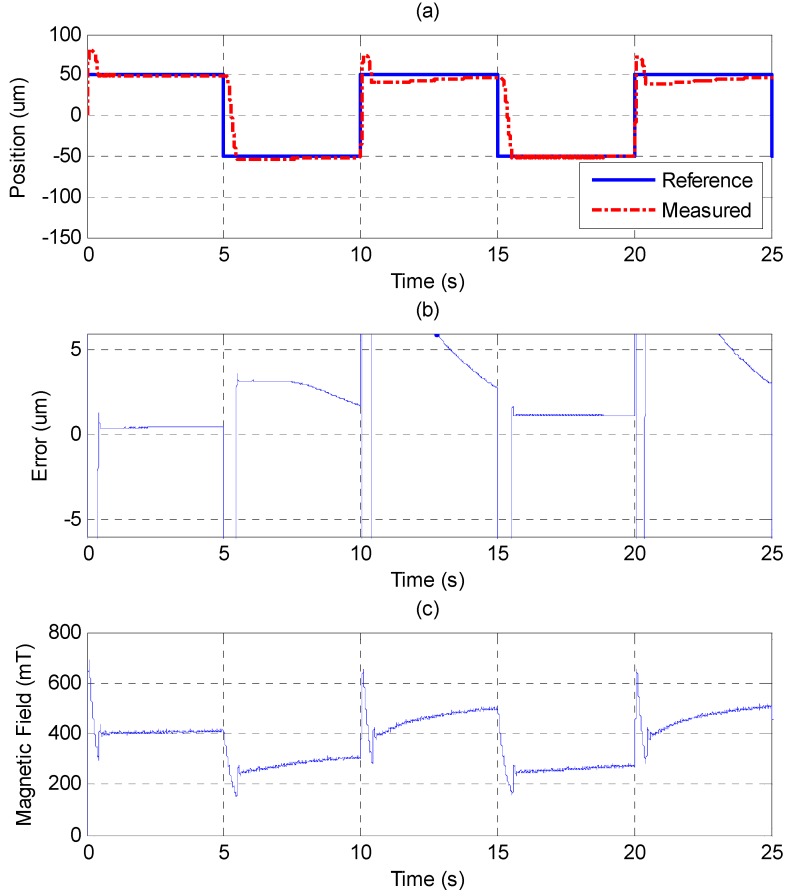
Step response of ±100 μm by PID controller with fast transient response: (**a**) position control response; (**b**) control error; (**c**) control input (magnetic field).

In the experiments, −50 μm and 50 μm are given as the tracking positions for the MSM actuator, and *u_o_* is 0.31 V, providing the median position in the trajectory. Figure 8 and Figure 9 show the experimental results derived from different PID control parameters. The experimental results show the nonlinear property of the hysteresis phenomenon. Tuning the parameters of the PID controller by the Ziegler-Nichols method [26] cannot track both of the given positions well. Figure 8 shows that the MSM actuator has tracking ability up to 50 μm of elongation, but it causes a large overshoot when it comes back to −50 μm. In Figure 9, the parameters of the PID controller are tuned to increase the system response of the MSM actuator. The MSM actuator can contract to −50 μm without overshooting. However, the fast response causes overshoot when the MSM actuator elongates to 50 μm. The large overshoot should be avoided because the MSM actuator is used as a precise micro positioning actuator. The position control response of the PID controller shows that the MSM actuator can only track one position without overshooting. If 50 μm is achieved without overshooting, then −50 μm would have a large overshoot, and *vice versa*.

**Figure 10:**
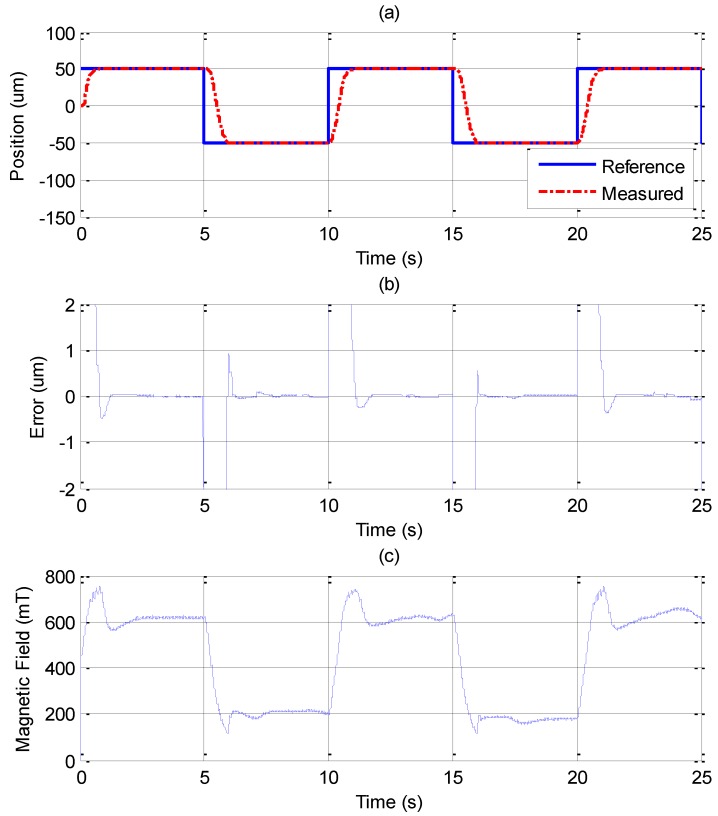
Step response of ±100 μm by MFSMC: (**a**) position control response; (**b**) control error; (**c**) control input (magnetic field).

In Figure 10, the MFSMC is used to control the MSM actuator. Compared to the PID controller, the MFSMC has noticeably better results. The control parameters of MFSMC for step response, including the offset control input *u_o_*, the fuzzy sliding surface *σ*, the membership functions *M*(*σ*) and *M*(*u*), the boundary layer of the fuzzy sliding surface Φ, and the scaling factor of the control input *G_u_* are given in Table 3. The MSM actuator with MFSMC can reach the reference position well without overshooting, which is a better performance than that of the PID controller at both of the reference positions. The steady-state error can reach the maximum resolution of 20 nm.

**Table 3:** Control parameters of MFSMC.

	Step Response	Sine Wave Trajectory
*u_o_*	3.1	3.1
*σ*	$8e+\overset{\cdot }{e}$	$10e+\overset{\cdot }{e}$
Φ	0.33	1
*G_u_*	6	2.3
*M*(*σ*)	{−1, −0.67, −0.33, 0, 0.33, 0.67, 1}	{−1, −0.67, −0.33, 0, 0.33, 0.67, 1}
*M*(*u*)	{−6, −4, −2, 0, 2, 4, 6}	{−2.30, −1.53, −0.77, 0, 0.77, 1.53, 2.30}

### 4.3. Sinusoid Trajectory Control

**Figure 11:**
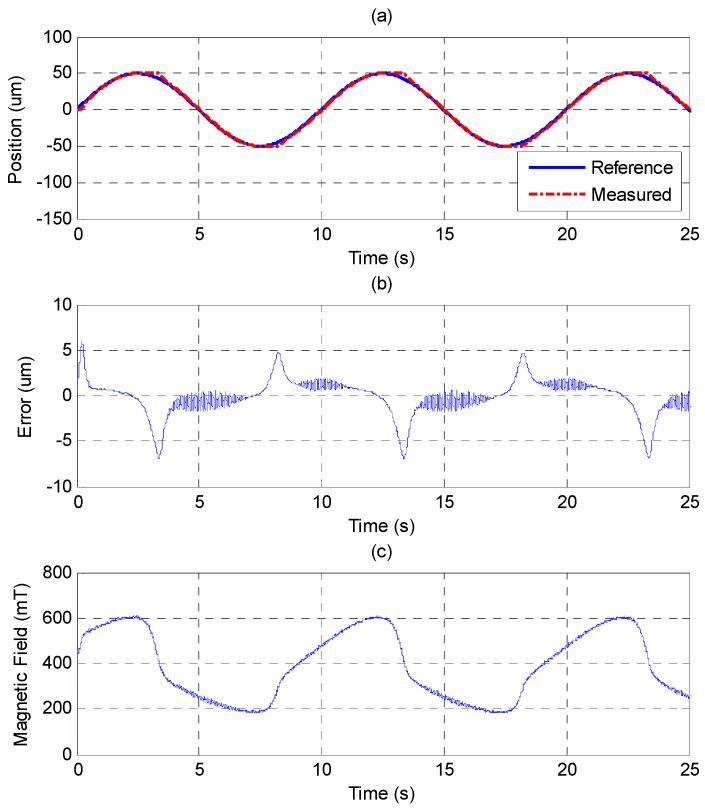
Tracking control of sinusoid trajectory with 0.1 Hz frequency and 50 μm amplitude by PID control: (**a**) position control response; (**b**) control error; (**c**) control input (magnetic field).

**Figure 12:**
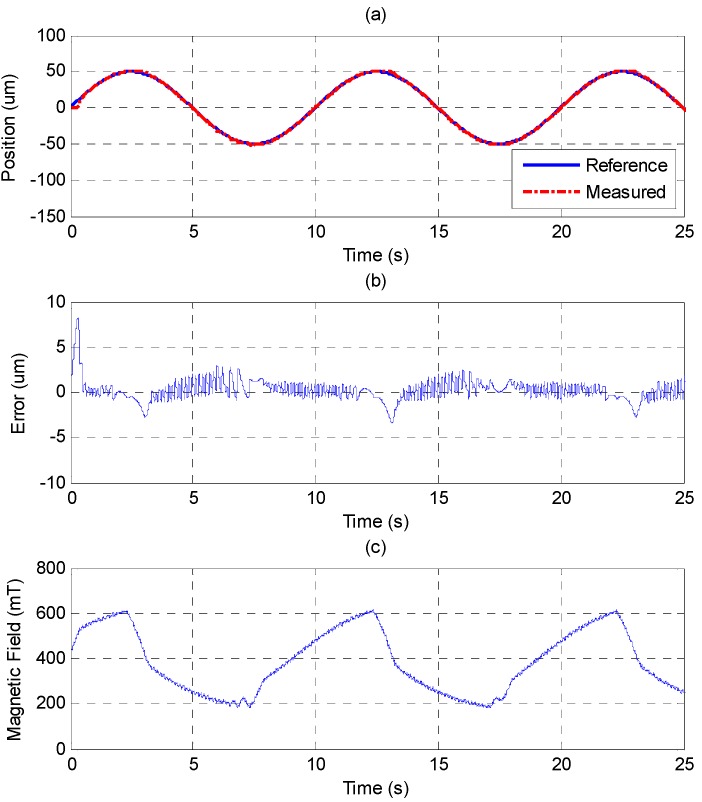
Tracking control of sinusoid trajectory with 0.1 Hz frequency and 50 μm amplitude by MFSMC: (**a**) position control response; (**b**) control error; (**c**) control input (magnetic field).

The sinusoid reference trajectory is set as 0.1 Hz of frequency and 50 μm of amplitude. Figure 11 shows that the PID controller cannot track the reference well as the MSM actuator changes the direction due to the effect of the friction force. In comparison with Figure 11, Figure 12 shows that the MFSMC performs better because it has the ability to switch signals quickly on a sliding surface, which improves the performance of tracking and reduces the influence of friction. The control parameters of MFSMC for sinusoid trajectory response, including the offset control input *u_o_*, the fuzzy sliding surface *σ*, the membership functions *M*(*σ*) and *M*(*u*), the boundary layer of the fuzzy sliding surface Φ, and the scaling factor of the control input *G_u_* are given in Table 3.

**Figure 13:**
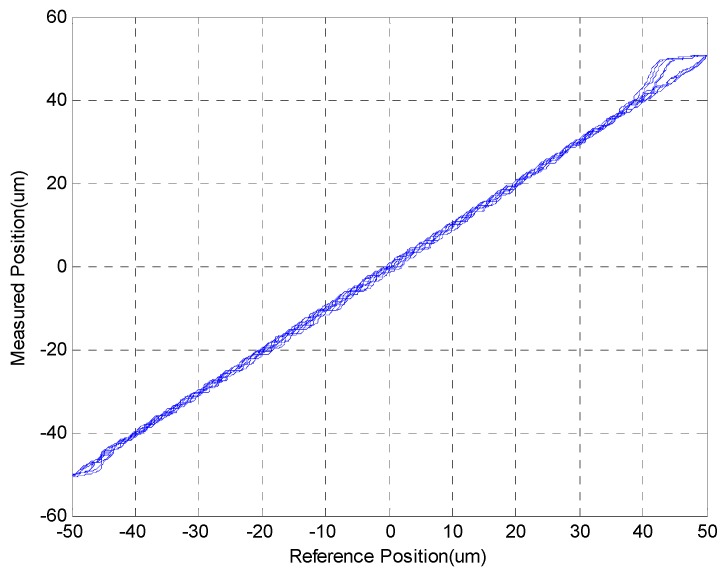
Linear relationship between reference position and measured position in sinusoid trajectory controlled by MFSMC for MSM actuator.

In Section 4.1, it was shown that the MSM actuator has a serious hysteresis phenomenon. The MSM actuator can be linearized by using the closed-loop control of MFSMC, as shown in Figure 13. The hysteresis can be improved to about 6% of full stroke. The slight effect of the friction force also exists as the MSM actuator changes the motion direction.

**Figure 14:**
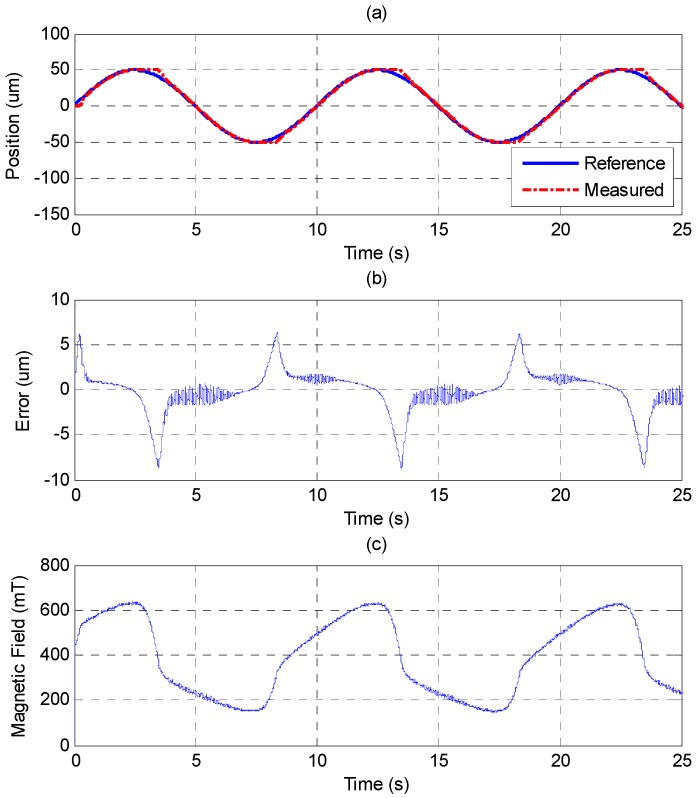
Tracking control of sinusoid trajectory with 100 g load, 0.1 Hz frequency and 50 μm amplitude by PID control: (**a**) position control response; (**b**) control error; (**c**) control input (magnetic field).

**Figure 15:**
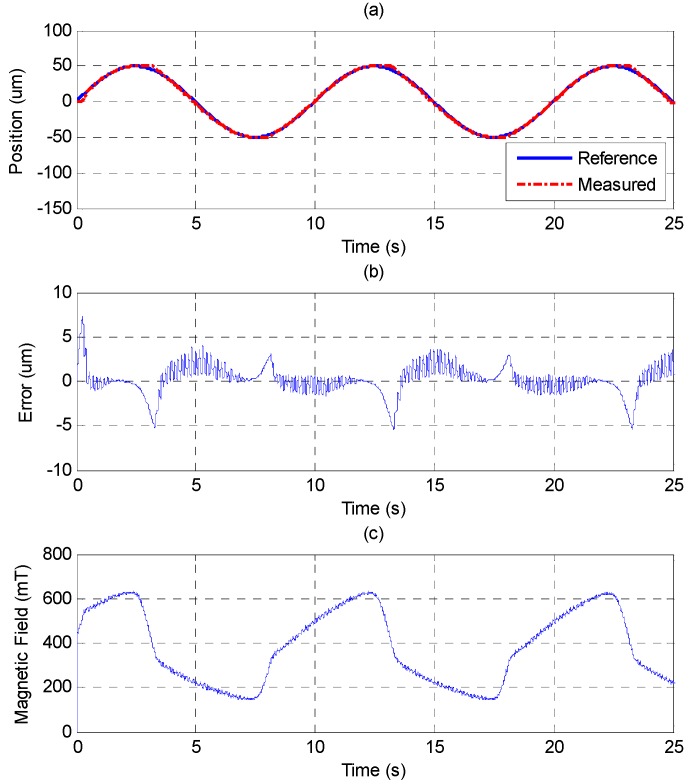
Tracking control of sinusoid trajectory with 100 g load, 0.1 Hz frequency, and 50 μm amplitude by MFSMC: (**a**) position control response; (**b**) control error; (**c**) control input (magnetic field).

In Figure 14 and Figure 15, the performance of PID control and MFSMC in sinusoid tracking with loading of 100 g, 0.1 Hz of frequency and 50 μm of amplitude are demonstrated. Due to the loading, the MSM actuator has larger inertia mass, lower response speed, and larger friction force. In Figure 14, it is hard for the PID controller to drive the MSM actuator as the motion direction is changed. However, the MFSMC better overcomes the system condition variations, as shown in Figure 15.

**Figure 16:**
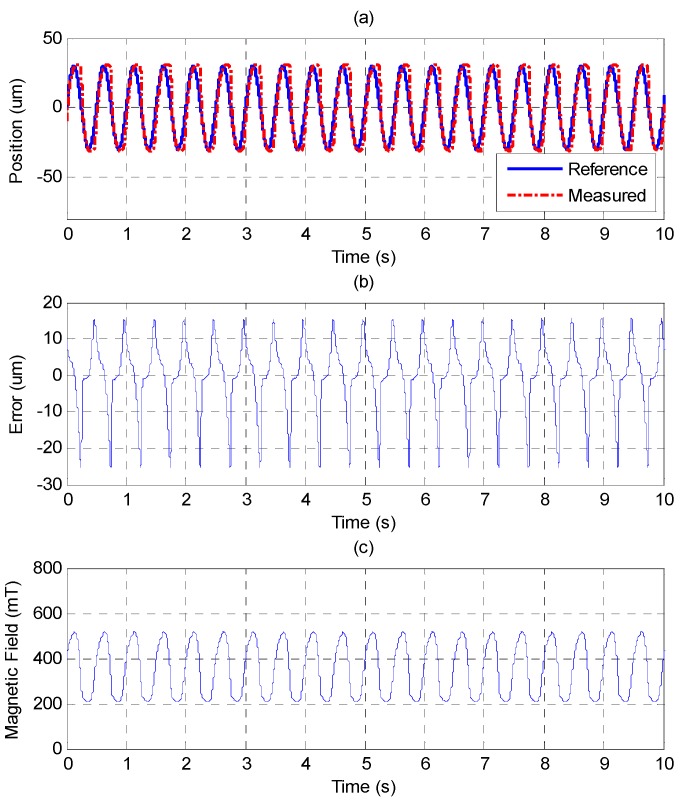
Tracking control of sinusoid trajectory with 2 Hz frequency and 30 μm amplitude by PID control: (**a**) position control response; (**b**) control error; (**c**) control input (magnetic field).

**Figure 17:**
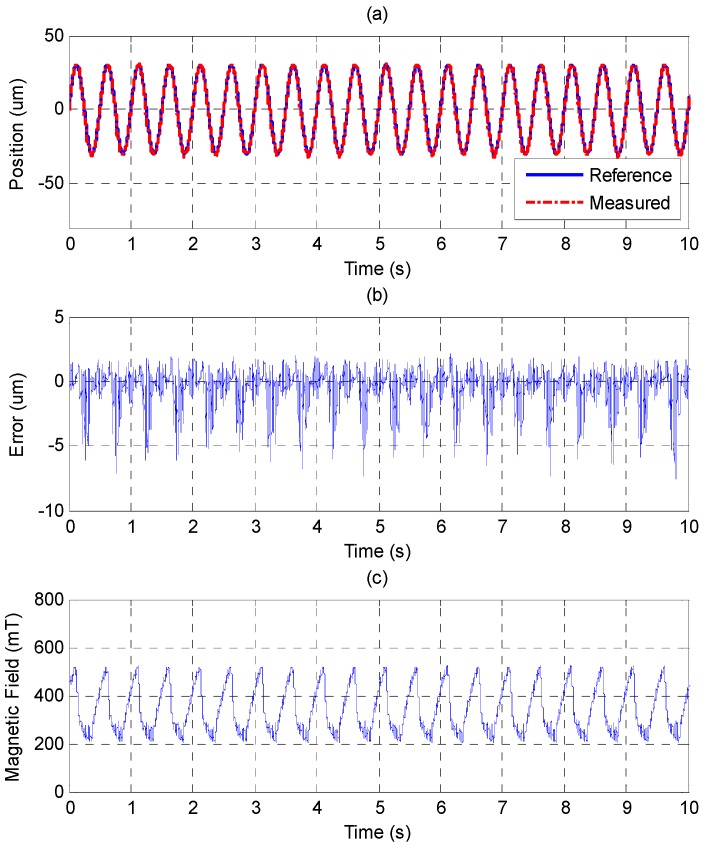
Tracking control of sinusoid trajectory with 2 Hz frequency and 30 μm amplitude by MFSMC: (**a**) position control response; (**b**) control error; (**c**) control input (magnetic field).

The experimental results of higher frequency of 2 Hz and 30 μm of amplitude by PID and MFSMC are shown in Figure 16 and Figure 17. The control response difference between the two controllers becomes more obvious. The MFSMC can maintain a controlled performance. However, the control error of the PID controller increases.

## 5. Conclusions

In this paper, we exploited the characteristics of MSM alloys and used them to set up a test rig of the MSM actuator. A perpendicular magnetic field elongates the MSM element proportionally with the magnetic field strength. The return motion is either by spring force or by a parallel magnetic field. For control purposes, continuous signals were provided to drive the MSM alloys instead of on-off signals to examine the characteristics of the MSM alloys.

Different input signals caused different hysteresis loops. In the first experiment, the relationship between the magnetic field and the position was shown, and the wide hysteresis phenomenon of MSM actuator was observed. The hysteresis phenomenon of the MSM actuator increases the difficulty of accurate position control. 

In the next experiment, the closed-loop control was used to achieve positioning control. For that, the modified fuzzy sliding mode control (MFSMC) was proposed. The MFSMC and the PID controller were implemented and compared. The MFSMC can perform better than the PID controller in the step response and the sinusoid trajectory tracking response under different frequency, loading, and amplitude. 
